# Informing Eating Disorder Support Through Lived Experience

**DOI:** 10.1177/23743735251346617

**Published:** 2025-05-30

**Authors:** Samantha H. Irwin, Abigail McCluskey, Sunny Y. Dong, Isra Amsdr, Anne Marie Portelli, Carla Southward, Britt Udall, Francine Buchanan, Matt Carwana, Nadia Roumeliotis, POPCORN Executive Committee

**Affiliations:** 1 7979 The Hospital for Sick Children, Toronto, ON, Canada; 2 Research Institute, British Columbia Children's Hospital, Vancouver, BC, Canada; 3 Department of Pediatrics, University of British Columbia, Vancouver, BC, Canada; 4 CHU Sainte-Justine Research Center, Montreal, QC, Canada; 5 Department of Pediatrics, Faculty of Medicine, University of Montreal, Montreal, QC, Canada

**Keywords:** patient engagement, patient perspectives, lived experience, mental health, pediatrics

## Abstract

An increase in eating disorder hospitalizations was observed amongst Canadian adolescents during COVID-19 public health restrictions. To help understand why this may have occurred, youth with lived experience of an eating disorder share their interpretations of these findings. This article, written by youth patient partners, provides insights into how unpredictable changes to daily routines and health system challenges during the COVID-19 pandemic might have influenced eating disorder hospitalizations. The increase in hospitalizations during the pandemic, combined with our lived experience advisory, underscores gaps in current approaches to supporting young people with eating disorders. We provide suggestions for clinicians, researchers, and policymakers stemming from our patient experiences in hopes that equitable, accessible, and patient-centered support can be prioritized to improve eating disorder-related care. This collaboration establishes a precedent for incorporating the voices of youth patient partners to better translate and mobilize research. These reflections serve as an example of how youth patient partner involvement can inspire future research, healthcare, and policy to advance care for those impacted by eating disorders.

## Introduction

A recent Canadian study, led by Pediatric Outcome imProvement through COordination of Research Networks (POPCORN), found a significant rise in female adolescent eating disorder (ED) hospitalizations during COVID-19 public health restrictions.^1^ To interpret these findings, POPCORN engaged our team of youth patient partners. A further aim of this project was to amplify voices of youth with lived experience of EDs to enhance the relevance and benefit of the original study.^2^

## Practical Perspectives

This section presents themes from discussions with youth and parents of youth with lived experiences of EDs during the COVID-19 pandemic (methods in Appendix). Our consultation focused on increases in ED hospitalizations for Canadian youth during pandemic public health restrictions.^1^ Emerging explanations for this finding included loss of control, disrupted care transitions, inaccessible or noninclusive ED treatment, perceived stigma from clinicians, the harmful influence of social media during periods of isolation, and reduced treatment capacity. Our patient perspectives highlight links between these considerations to inform future ED research, treatment, and policy.

***Lack of Control Can Worsen ED Thoughts and Behaviors.*** We were unsurprised that the pandemic was associated with more youth ED hospitalizations.^1,3,4^ Disruptions to routines and fluctuating public health restrictions left us feeling without control, worsening our ED symptoms. As one youth patient partner expressed:

*I feel anxious when my external world is chaotic, as it was during the pandemic. I chose dieting and exercise as my coping strategies because they provided control.*

Losing access to fulfilling activities (eg, school, extracurricular events, and social events) reduced our motivation for ED recovery, consistent with other research.^3^ Losing autonomy over daily activities increased our need to cope, often achieved through ED behaviors. Conversely, brief returns to “life as usual” when restrictions lifted provided a familiar routine and sense of control, protecting against our ED symptoms.

***Transitioning to Adult Services Can Be a Barrier in ED Recovery.*** Fewer ED hospitalizations among young adults compared to adolescents^1^ during COVID-19 might be due to young adults transitioning out of pediatric services. Members of our group experienced inconsistent care transfers and unsupportive interactions with clinicians who were uninformed about EDs or pessimistic about recovery.^5–7^ As members of our patient partner group approached adulthood, many received warnings from treatment providers about adult ED services:

*My clinicians warned me about the inadequacies of adult eating disorder care. I was hesitant to seek support after turning 17, as I was told that they would not be able to help me.*

Some young adults avoided seeking care because of previous stigma from clinicians. For example, being told we were not “sick enough” based on weight criteria without considering other symptoms, or being blamed for repeated treatment attempts. These concerns are not unique to our group and are captured by other research examining barriers to ED care.^6^

Higher increases in ED hospitalizations for adolescents compared to young adults^1^ might also relate to greater parental involvement in ED care when patients are younger.^4^ Adolescents often live with caregivers who can monitor ED behaviors more closely, leading to earlier intervention. Differences in ED hospitalization rates for the two age groups might therefore reflect protective factors (eg, more confidence in ED care for younger patients, parental involvement).

***Gender Is Not Appropriately Considered in ED Spaces.*** Young males accounted for only 10% of ED hospitalizations during COVID-19,^1^ mirroring our experiences. ED symptoms in boys and young men might present or be assessed differently, leading to missed diagnoses due to assumptions that disordered eating is uncommon in men.^8^ EDs in young males might therefore be underrepresented in hospitalization data. As such, increasing recognition of gender diversity in ED treatment and research spaces is crucial.

***Social Media Can Reinforce ED Behaviors.*** Restrictions on in-person interactions increased our social media use during the pandemic. Seeking social connectedness online, we encountered overwhelming diet culture content. Social media can perpetuate ED behaviors via social comparison, thin-ideal internalization, and self-objectification.^9^ Our group of patient partners reported that in-app features to restrict this harmful content were insufficient. Many of us had to delete social media accounts entirely, further limiting meaningful social connection during an already isolating time.

***Reduced ED Services and Focus on Weight Resulted in Insufficient Care*.** Access to ED care was reduced during public health restrictions, with many services being shut down indefinitely. For those who could access outpatient care during the pandemic, these services were only offered virtually. This meant no physical health exams despite remote therapeutic support:

*Without in-person care, no one realized how quickly my physical health deteriorated. This led to an unexpectedly long hospital admission for medical stabilization.*

In-person medical monitoring is a cornerstone of ED treatment,^10^ and the absence of in-person care during public health restrictions posed a considerable dilemma. Without comprehensive assessment of both mental and physical health, members of our group were provided insufficient outpatient support, making the need for in-person monitoring apparent.

Wait lists for other services (eg, inpatient programs) were lengthy and seemed to be triaged by weight loss rather than other core ED symptoms like emotional distress. After waiting months for treatment, brief inpatient admissions focusing on weight restoration did not provide us with tools to manage ED symptoms at home. We then experienced cycles of hospital readmission or avoided future care for fear of being labeled “treatment resistant” or feeling hopeless about recovery.^6^

By contrast, clinicians misdiagnosed an ED in one patient partner who experienced weight loss due to another medical condition:

*I was hospitalized multiple times for a digestive condition but still diagnosed with an ED. Even after appropriate testing, the ED treatment team came to see me to get me to ‘just eat.’*

Our perception is that insufficient assessment and focus on weight can cause clinicians to mislabel the presence or severity of an ED. Without full evaluation and increased treatment capacity for both mental and physical ED symptoms, we are provided inadequate support which can negatively impact our health.

## Recommendations

We urge clinicians, scientists, and policymakers to consider the insights and recommendations stemming from our lived experience of EDs to improve future research, policy, and care:

1. ***Prioritize Autonomy in ED Recovery and Daily Life.*** Allowing young people with EDs autonomy over treatment and daily routines can help us feel in control, reducing the likelihood of turning to harmful coping mechanisms.^3^ We encourage care providers to prioritize patient self-determination when providing ED support. This could involve treatment plans developed collaboratively with the patient, which also encourage activities that give life meaning beyond the ED.
2. ***Involve Families in Treatment and Promote Continuous Care During Transition Ages.*** We recognize the benefit of parents and caregivers supporting younger ED patients.^4,6^ Meaningful engagement of families throughout treatment might improve ED outcomes, as could better collaboration between pediatric and adult service providers during ED care transitions.^5^ We also advise ED care providers to be recovery-oriented when speaking with patients who are graduating to adult services. Clinicians should share the belief that recovery is always possible irrespective of the patient's age, unique challenges, or treatment options.
3. ***Challenge Attitudes and Stereotypes Toward EDs.*** We encourage care providers to move away from common ED misconceptions and stereotypes.^5,6^ EDs can present within all ages, races, ethnicities, sexes, genders, and body types. ED screening and referrals to appropriate care should be conducted regardless of these factors, and irrespective of the clinician's experience with or beliefs about EDs.
4. ***Regulate Harmful Social Media Content.*** Social media platforms should be held accountable to enforce stricter censorship of diet and ED-related content. In-app features should be improved to better allow individuals to restrict triggering content. Policies should also be implemented to eliminate social media algorithms that target vulnerable youth with harmful dieting or ED-related content.
5. ***Maintain Consistent, Accessible and Fulsome ED Care.*** ED services must be equipped to provide ongoing, reliable, and accessible treatment that provides support for both mental and physical symptoms of EDs. We recommend a combination of in-person and virtual care to ensure comprehensive support.^10^ Treatment capacity must also be increased to meet the sustained rise in youth who require ED care.^1^ We strongly advocate for expanded services where access to treatment is not primarily based on weight, and psychological and behavioral symptoms should be prioritized. If emotional distress related to ED symptoms is supportively addressed before physical consequences arise, we believe patients might not be put at risk of medical instability.
6. ***Meaningfully Integrate and Amplify Lived Experience.*** There is tremendous mutual benefit to care providers, researchers, and patients when individuals with lived experience are engaged.^2^ We encourage continued collaboration with individuals who have lived experience, as our voices are essential in creating more effective, compassionate care.

## Conclusions

Gaps in current approaches to supporting young people with EDs were magnified by COVID-19 hospitalization trends and confirmed through this patient partner collaboration. Our lived experiences highlight the urgent need for more inclusive, accessible, and comprehensive ED care. By incorporating our patient perspectives into research, treatment, and policy, we can inform equitable, patient-centered care to improve ED outcomes.

## Supplemental Material

sj-docx-1-jpx-10.1177_23743735251346617 - Supplemental material for Informing Eating Disorder Support Through Lived Experience Supplemental material, sj-docx-1-jpx-10.1177_23743735251346617 for Informing Eating Disorder Support Through Lived Experience by Samantha H. Irwin, Abigail McCluskey, Sunny Y. Dong, Isra Amsdr, Anne Marie Portelli, Carla Southward, Britt Udall, Francine Buchanan, Matt Carwana, Nadia Roumeliotis and POPCORN Executive Committee in Journal of Patient Experience

## Appendix

Pediatric Outcome imProvement through COordination of Research Networks (POPCORN) brings together researchers, clinicians, decision-makers, and patient partners to form a pan-Canadian pediatric research platform and answer important questions about youth health. While initially created in response to the COVID-19 pandemic, the platform provides the infrastructure for future projects dedicated to improving health outcomes for youth. POPCORN is made up of the leaders of four national research networks and supported by a coordinating center along with teams of experts on how to collect, share, and analyze data across projects. To ensure patient and family perspectives are integrated across the network, youth and parent partners are members of the leadership team and support research activities.

Youth and parents (of youth) with a history of EDs, anxiety, and/or self-harm were recruited across Canada to participate in consultation with POPCORNresearchers regarding a population-based study using administrative health data. Youth and parents were recruited via social media channels, websites, and word of mouth. Seven youth (age range 19-26) and one parent of youth with a history of accessing ED-related during the COVID-19 pandemic participated in a discussion group with the original research team (see Appendix Table 1). The ED services patient partners accessed during the COVID-19 pandemic included community-based care, public out-patient and day-treatment programs, private counseling and/or medical monitoring services, emergency department visits, and inpatient hospitalizations. Group members resided across Canada and represented a diversity of ethnicities and cultural backgrounds:

**Table 1.** Demographics of Youth and Parents Participating in Group Discussion.

Youth/Parent	Age	Province	Ethnic or Cultural Background
Youth 1	21	New Brunswick	Arabic
Youth 2	23	Ontario	Chinese
Youth 3	26	Ontario	Caucasian
Youth 4	19	British Columbia	Caucasian
Youth 5	24	Ontario	Caucasian
Youth 6	21	Ontario	Caucasian
Youth 7	21	Saskatchewan	Latin American/Caucasian
Parent	Unreported	Ontario	Caucasian

Data on the rate of hospitalizations in Canada for EDs and other mental health conditions before and during COVID-19 public health restrictions were presented to the group. Patient partners were guided in a collaborative discussion to share their pandemic experiences and interpretations of the hospitalization data. The rich discussion with youth and parents led to an aim of sharing such perspectives in a meaningful way. This catalyzed the current youth patient partner-led commentary, authored by a subset of members from the patient partner group. All patients and family members who engaged in this discussion agreed to their perspectives being shared as part of this piece even if they are not included as authors.
