# Supplementary material for: Informing Eating Disorder Support Through Lived Experience
Source: J Patient Exp. 2025 May 30;12:23743735251346617. doi: 10.1177/23743735251346617 (PMC12126641; doi:10.1177/23743735251346617)
Supplement: sj-docx-1-jpx-10.1177_23743735251346617 - Supplemental material for Informing Eating Disorder Support Through Lived Experience [file sj-docx-1-jpx-10.1177_23743735251346617.docx]

**Appendix**

[Name of research network removed for anonymity] is a network that brings together researchers, clinicians, decision-makers, and patient partners to form a pan-Canadian pediatric research platform and answer important questions about youth health. While initially created in response to the COVID-19 pandemic, the platform provides the infrastructure for future projects dedicated to improving health outcomes for youth. The [name of research network removed for anonymity] platform is made up of the leaders of four national research networks and supported by a coordinating center along with teams of experts on how to collect, share, and analyze data across projects. To ensure patient and family perspectives are integrated across the network, youth and parent partners are members of the leadership team and support research activities.

Youth and parents (of youth) with a history of eating disorders, anxiety, and/or self-harm were recruited across Canada to participate in consultation with [name of research network removed for anonymity] researchers regarding a population-based study using administrative health data. Youth and parents were recruited via social media channels, websites, and word of mouth. Seven youth (age range 19-26) and one parent of youth with a history of accessing ED-related during the COVID-19 pandemic participated in a discussion group with the original research team (see Appendix Table 1). The ED services patient partners accessed during the COVID-19 pandemic included community-based care, public out-patient and day-treatment programs, private counseling and/or medical monitoring services, emergency department visits, and inpatient hospitalizations. Group members resided across Canada and represented a diversity of ethnicities and cultural backgrounds:

Table 1. Demographics of youth and parents participating in group discussion

| **Youth/Parent** | **Age** | **Province** | **Ethnic or Cultural Background** |
| --- | --- | --- | --- |
| Youth 1 | 21 | New Brunswick | Arabic |
| Youth 2 | 23 | Ontario | Chinese |
| Youth 3 | 26 | Ontario | Caucasian |
| Youth 4 | 19 | British Columbia | Caucasian |
| Youth 5 | 24 | Ontario | Caucasian |
| Youth 6 | 21 | Ontario | Caucasian |
| Youth 7 | 21 | Saskatchewan | Latin American/Caucasian |
| Parent | Unreported | Ontario | Caucasian |

Data on the rate of hospitalizations in Canada for EDs and other mental health conditions before and during COVID-19 public health restrictions were presented to the group. Patient partners were guided in a collaborative discussion to share their pandemic experiences and interpretations of the hospitalization data. The rich discussion with youth and parents led to an aim of sharing such perspectives in a meaningful way. This catalyzed the current youth patient partner-led commentary, authored by a subset of members from the patient partner group. All patients and family members who engaged in this discussion agreed to their perspectives being shared as part of this piece even if they are not included as authors.
